# CCL2/CCR2 Signalling in Mesenchymal Stem/Progenitor Cell Recruitment and Fracture Healing in Mice

**DOI:** 10.1111/jcmm.70300

**Published:** 2024-12-25

**Authors:** Rahasudha Kannan, Amy J. Koh, Robert N. Kent, Kaira Bhutada, Fatima Wasi, Leon Wagner, Kenneth Kozloff, Brendon M. Baker, Hernan Roca, Laurie K. McCauley

**Affiliations:** ^1^ Department of Periodontics and Oral Medicine University of Michigan School of Dentistry Ann Arbor Michigan USA; ^2^ Department of Biomedical Engineering University of Michigan Ann Arbor Michigan USA; ^3^ Department of Orthopaedic Surgery University of Michigan Ann Arbor Michigan USA; ^4^ Department of Pathology University of Michigan Medical School Ann Arbor Michigan USA

**Keywords:** C‐C motif chemokine ligand 2, efferocytosis, fracture healing, osteoimmunology, osteoprogenitor

## Abstract

Macrophage efferocytosis (clearance of apoptotic cells) is crucial for tissue homeostasis and wound repair, where macrophages secrete factors that promote resolution of inflammation and regenerative signalling. This study examined the role of efferocytic macrophage‐associated CCL2 secretion, its influence on mesenchymal stem/progenitor cell (MSPC) chemotaxis, and in vivo cell recruitment using *Ccr2* ^−/−^ (KO) mice with disrupted CCL2 receptor signalling in two regenerative models: ossicle implants and ulnar stress fractures. Single cell RNA sequencing and PCR validation indicated that efferocytosis of various apoptotic cells at bone injury sites (osteoblasts, pre‐osteoblasts, MSPC) upregulated CCL2. CCL2 gradients enhanced MSPC migration through type I collagen matrices. In vivo, MSPC (LepR^+^) infiltration was significantly reduced while macrophage (F4/80^+^) infiltration increased in KO ossicle implants versus WT. In ulnar stress fractures, micro‐CT revealed increased mineralized callus incidence in CCR2 KO male mice 5 days post injury (dpi) versus WT. By 7‐dpi callus fractional bone volume, trabecular thickness, and bone mineral density were increased versus WT. Immunohistochemistry of mice 5‐dpi confirmed an increase in callus area (including soft tissue); however, the percent of osteoprogenitors (%Osx^+^) within the callus was not different. These findings suggest that CCL2 differentially impacts MSPC recruitment depending on bone wound healing model.

## 1 Introduction

Macrophage efferocytosis, or phagocytic clearance of apoptotic cells (ACs), is a crucial process for wound repair and restoration of tissue homeostasis [1]. Deficiency in clearing ACs has been implicated in aberrant wound healing and a variety of pathologies including diabetes, rheumatoid arthritis, neurodegenerative diseases, and lung inflammation [2, 3]. Macrophage efferocytosis is a necessary component of bone homeostasis [4], and impaired efferocytosis has been shown to negatively impact osseous healing outcomes in various injury and disease models [5, 6, 7, 8, 9, 10]. Many studies have demonstrated a crucial role for macrophages in bone development and repair, but specific contributions of macrophages through their efferocytic activity are still actively being studied [11, 12, 13, 14, 15].

These highly plastic, phagocytic white blood cells are generally believed to shift towards more reparative activity following efferocytosis. Part of this process involves the secretion of factors that facilitate the resolution of inflammation and regeneration, such as TGF‐β1 and IL‐10, while suppressing pro‐inflammatory cytokines including TNF, IL‐1, and IL‐12 [1, 2, 4, 16, 17, 18, 19, 20]. These factors influence different phases of wound repair, from stem/progenitor cell recruitment to differentiation.

Michalski et al. previously showed that macrophage engulfment of apoptotic bone marrow stromal cells (BMSC) upregulated TGF‐β1 and C‐C motif chemokine ligand 2 (CCL2), two factors known to influence the bone remodelling process [18]. Among the factors secreted by efferocytic macrophages, CCL2 is likely central to macrophage‐mediated osseous repair. Most commonly viewed as a myeloid cell chemoattractant, CCL2 is actually a pleiotropic factor that also influences leukocyte activity and other facets of myeloid cell behaviour, including adhesion, survival, and maturation/differentiation [21]. Numerous studies have also implicated CCL2 in mesenchymal stem cell recruitment and wound healing [22, 23, 24]. Taken together, these studies suggest CCL2 plays a role in bone regeneration beyond macrophage recruitment. While some in vivo studies have shown impaired healing after bone injury in CCL2 receptor (CCR2) antagonism or genetic knock‐out models [22, 25, 26], other studies have shown no impact on bone repair [27, 28]. Although CCL2 has been studied for decades, the nuances of its role in bone repair are still incompletely understood.

In this study CCL2's roles in mesenchymal stem/progenitor cell (MSPC) recruitment and osseous wound healing were investigated in vitro and in two distinct in vivo experimental models. While the ossicle implantation focused on in vivo macrophage and MSPC migration isolated from the bone microenvironment, the ulnar stress fracture model examined MSPC recruitment in the context of early bone repair. Taken together, the results shows that CCL/R2 signalling influences both MSPC recruitment and early stress fracture callus formation.

## 2 Materials and Methods

### 2.1 Animals and Cell Culture

All animals were maintained in accordance with institutional animal care and use guidelines, and experimental protocols were approved by the Institutional Animal Care and Use Committee of the University of Michigan. C57BL/6J (The Jackson Laboratory, Bar Harbour, ME) mice were bred in house and used for in vitro experiments. C57BL/6 mice, CCR2 KO (*Ccr2* ^−/−^) [29] mice, LepRiTom C57BL/6 mice (generously provided by Deneen Wellik) [30], and LepRiTom CCR2 KO mice were used for in vivo studies. Both wildtype C57BL/6 (WT) and CCR2 KO mice were bred with LepR‐Cre Rosa‐tdTomato expression to track and evaluate mesenchymal stem/progenitor cell infiltration.

Primary bone marrow flush was collected from tibiae and femurs (4 bones per mouse) of 4‐ to 6‐week old C57BL/6J mice [18]. Bone marrow‐derived macrophages (MΦs) were differentiated in vitro from bone marrow flush in α‐MEM medium (10% FBS, 1% Pen/Strep) with murine M‐CSF (30 ng/mL, BioLegend, San Diego, CA, USA; 576,406) for 5–7 days. Half the volume of media was removed and replaced with an equivalent amount of fresh media (30 ng/mL MCSF for the *total* volume of media) on Day 3; a full media change was performed on Day 4, and if needed on Days 5–6. Macrophages were plated at 5 × 10^5^ cells/well in 12‐well plates (for flow cytometry) and 5 × 10^5^ cells/well in 6‐well plates (for qRT‐PCR and ELISA). Bone marrow stromal cells (BMSCs) were differentiated in vitro from bone marrow flush in α‐MEM medium (20% FBS, 1% Pen/Strep) with dexamethasone (10 nM, Sigma, St. Louis, MO, USA; D8893) for 6 days and used between passage 1–2. Media (5 mL) was added on Day 3; half the volume of media was removed and an equivalent amount of fresh media was added on Day 4; and a full media change was performed on Day 5.

Primary neonatal calvarial osteoblasts (OB) were collected from C57BL/6J mouse pups (< 2 weeks old). Calvaria were isolated and washed three times in 1× PBS, then plain α‐MEM, followed by incubation in digestion media (α‐MEM, 2 mg/mL collagenase A, 1× trypsin) in a 37°C rocker (200–240 rpm) for 10, 20 and 60–90 min, with digestion media replaced between each incubation. After the final digest, calvaria and digestion media were filtered through a 40 μm cell strainer, centrifuged (4 min, 500×*g*), and washed 1× in complete α‐MEM before plating into 150 mm tissue culture plates for expansion. Murine pre‐osteoblastic cells from the MC3T3‐E4 (MC4) murine cell line (passage 11–15) were expanded in α‐MEM medium (10% FBS, 1% Pen/Strep) for 4–6 days.

### 2.2 Phagocytosis and Efferocytosis Assays

MC4 (passage 12–16), OB (passage 1–2), and BMSC (passage 1–2) were plated at ~40,000 cells/cm^2^ the day before starting efferocytosis experiments. For flow cytometry, cells were stained with 2 μM CellTrace CFSE (Invitrogen, Waltham, MA, USA; C34554) per manufacturer instructions before apoptosis induction. Cells were rinsed with 1× PBS and exposed to UV light (Fisher, Transilluminator F8TIV‐816) in 1× PBS for 30 min to induce apoptosis. Apoptotic cells (ACs) were then incubated in their respective growth media for 1 h at 37°C, harvested using a cell scraper, filtered through a 40 μm cell strainer, counted via trypan blue exclusion (confirming cell death), and added to MΦ cultures (passage 1) at a 2:1 AC to MΦ ratio for 18 h (scRNA‐seq, qRT‐PCR, ELISA), or 5 and 25 h (FACS).

### 2.3 Flow Cytometry

At the termination of phagocytosis experiments, MΦs were washed twice in 1X PBS and collected in plastic cell culture tubes in FACS buffer (1× PBS, 2% FBS, 2 mM EDTA). After centrifugation (3–4 min, 1000×*g*, 4°C), MΦs were stained with 2 μL F4/80‐APC (BioLegend, 157,306) per 100 μL FACS buffer at room temperature for 20–30 min, then washed twice with FACS buffer. MΦs were then fixed in 2% formalin in FACS buffer. Samples were assessed for phagocytosis via flow cytometry (BD FACSAriaTM III, BD Biosciences, San Jose, CA, USA) for double labelled cells (APC^+^NileRed^+^ or APC^+^CFSE^+^) reflecting phagocytic engulfment.

### 2.4 Single Cell RNA Sequencing Experiment and Analysis

MΦs (passage 1) were co‐cultured with apoptotic OB (passage 1) for 18 h. OB were labelled with CFSE, and F4/80^+^CFSE^+^ efferocytic macrophages as well as control (non‐engulfing) macrophages were sorted by FACS and analysed by single‐cell sequencing using the 10× Genomics (Pleasanton, CA, USA) technology. Briefly, cells were combined with an RT‐mix and up to eight samples loaded in wells of a Chromium chip. Partitioning oil and beads (each containing a poly dT sequence, a 10× barcode sequence, and a unique molecular identifier sequence for each barcode), were also loaded on the chip. The 10× Chromium machine captures a single barcoded bead in an oil droplet, along with zero, one, or two cells according to a Poisson distribution, such that 60% of the loaded cells are captured. Following capture in the oil droplet, the mRNA was reverse transcribed into cDNA, and a portion was then amplified by PCR and fragmented. Adaptors were ligated and the product amplified again, using one of 96 indexing primer sets. Invitrogen Dynabeads and Beckman Coulter SPRI select beads were used to purify the product and ensure the final size range of the library to be sequenced was 200–800 bp. Cells (~10^4^/sample) were clustered based on transcriptional similarity using a *t*‐SNE algorithm for data visualisation and to compare control MΦ versus efferocytic MΦ engulfing apoptotic OBs. The analysis using the ‘cellranger aggregate’ software pipeline (10× Genomics) identified a distribution shift in the efferocytic cell transcriptome and the identification of clusters that predominantly grouped efferocytic or control macrophages.

### 2.5 qRT‐PCR

Total RNA was isolated from MΦ alone, ACs alone, and MΦ + AC co‐culture after 18 h using the Qiagen (Germantown, MD, USA) Rneasy Mini Kit. Reverse transcription was conducted using the High‐Capacity cDNA Reverse Transcription Kit (Thermo Fisher, Waltham, MA, USA; 4,368,814), and cDNA products were amplified and detected using TaqMan Gene Expression Master Mix (Thermo Fisher, 4,369,016) and TaqMan probes, including mouse *Ccl2* (Mm00441242_m1), mouse *Tgfb1* (Mm01178820_m1), and mouse *18* s RNA (Mm03928990_g1) as an endogenous control. Quantitative Real time PCR was analysed on ABI PRISM 7700 (Applied Biosystems, Waltham, MA, USA).

### 2.6 Enzyme‐Linked Immunosorbent Assay (ELISA)

Macrophage secreted CCL2 (MCP‐1) was quantified by RayBio Mouse ELISA, (RayBiotech Inc. Peachtree Corners, GA, USA; ELM‐MCP‐1). Macrophage conditioned media was collected from MΦ alone, AC alone, and MΦ + AC 18 h co‐culture, and diluted 8× in diluent B prior to ELISA.

### 2.7 In Vitro 3D Migration Assay

A previously established 3D migration assay [31] was used to evaluate BMSC migration to CCL2. The device contained two 300 μm channels, created by crosslinking a 3 mg/mL rat tail collagen type 1 gel (Corning, Corning, NY, USA; CB354249) around two parallel needles. Extraction of the moulding needles yielded two patent channels fully embedded in extracellular matrix that terminated in connected media reservoirs. BMSCs (passage 1) were seeded in one channel in full BMSC growth media (α‐MEM, 20% FBS, 1% Pen/Strep, 20 nM murine dexamethasone (Sigma)), and then switched to reduced‐serum BMSC growth media (10% FBS) the next day with subsequent media changes performed daily. BMSCs were allowed to migrate through the gel towards the other channel, which contained media with different concentrations of CCL2 (Peprotech, Cranbury, NJ; 250‐10). Devices were placed on a rocker inside a cell culture incubator to develop and maintain a chemokine gradient between the two channels.

On Day 7, BMSCs in the devices were stained with propidium iodide (1 mg/mL PI in BMSC media, 20 min, in incubator), fixed with 4% PFA (30 min), permeabilized (30–60 min), and stained with phalloidin‐AlexaFluor488 (1:1000) and Hoechst (1:1000) in 4% IgG‐free BSA solution (1 h). Devices were imaged on Zeiss LSM800 Confocal Microscope and migration was quantified using a custom MATLAB script, which computed the shortest distance of each nucleus to the user‐defined microchannel edge.

### 2.8 Ossicle Implantation Model

Ossicles were prepared and implanted in 4–6 week WT LepRiTom or CCR2 KO LepRiTom as established in a previous protocol [32]. Briefly, BMSCs (passage 1) from 4 to 6 week WT or CCR2 KO mice were seeded in Gelfoam/Surgifoam (New York, NY, USA) (2 million cells per 5 mm^3^) that were pre‐soaked in media, and implanted subcutaneously (2 ossicles/mouse) into 4–6 week WT LepRiTom and CCR2 KO LepRiTom mice, respectively. Ossicles were explanted after 4 weeks and processed for immunohistochemistry. Ossicle volume was calculated using an ellipsoid volume formula (V = π/6 × L × W × H), based on tumour volume calculation methodology [33].

### 2.9 Ulnar Stress Fracture Model

The ulnar loading model was used to induce stress fractures in adult (16 week) WT and CCR2 KO mice. Mice have completed their rapid skeletal growth phase at this age [34] and this model has been shown to induce non‐displaced fractures (stress fractures) via fatigue loading [35]. Mice were anaesthetised (1%–3% isofluorane) and forelimbs were loaded in axial compression across the flexed carpus and olecranon process into a custom built servo‐motor loader for monotonic loading, fatigue loading to complete fracture, or fatigue loading to stress fracture (partial, non‐displaced fracture). Fatigue loading was performed at 2 Hz under load‐control (i.e., same peak force for each load cycle). Force and displacement data were recorded using Labview 7.1 software (National Instruments, USA).

#### 2.9.1 Loading to Complete Fracture

For monotonic loading, the left forelimbs of mice (*n* = 3–5) were loaded to failure at a constant displacement of 0.5 mm/s to determine ultimate force required to fracture the bone. For fatigue loading, right forelimbs of the monotonically loaded mice (*n* = 3–5) were cyclically loaded to fracture at 80% of the force required to fracture their left forelimb. Mice were immediately sacrificed after fracture. The average force and displacement to complete fracture for each genotype were determined and used in subsequent fatigue loading for partial, non‐displaced fracture experiments.

#### 2.9.2 Loading to Stress Fracture (Partial, Non‐displaced Fracture)

Right forelimbs of mice were fatigue loaded (at a constant peak force = 80% of the force to complete fracture) until reaching the prescribed increase in axial displacement, corresponding to 60% of displacement to complete fracture. Mice were euthanized 3, 5, or 7 days post injury, and stress fractured right forelimbs, were harvested at time of euthanasia.

### 2.10 MicroCT And Histomorphometry

Dissected and fixed (4% PFA, 24–48 h) ulnae were scanned by ex vivo micro‐computed tomography at a 12 μm voxel size (Scanco μCT‐100, Schweiz, Switzerland) and assessed following established guidelines [36]. Ulnae were then stored in 70% ETOH, decalcified in 14% ethylenediaminetetraacetic acid (EDTA), embedded in OCT embedding medium, and sectioned at 18 μm at −20°C (Leica CM1850 Cryostat, Deerpark, IL, USA). Immunostaining of F4/80 (CST 70076T, Fisher A11034) and osterix (Abcam, Cambridge, UK; ab209484) was used to identify macrophages and osteoprogenitors, respectively. MSPC were identified via LepRiTom fluorescent labelling. Due to artifactual tearing of ulnae during cryosectioning, some samples were excluded from immunohistological analyses. Imaging was performed on a Zeiss 710 laser scanning microscope at 10× (0.5× zoom) and 20× (1× zoom). LepR, F4/80 and OSX fluorescence per region of interest (ROI), such as ossicle or callus, were quantified using a custom MATLAB script or Image J (NIH) [37].

### 2.11 Statistics

Statistical analyses were performed using GraphPad Prism 10 (GraphPad Software, version 10.2.3, San Diego, CA, USA) as specified in figure legends.

## 3 Results

### 3.1 Efferocytic Macrophages Engulfing Apoptotic Bone Cells Upregulated CCL2 Expression

**FIGURE 1:**
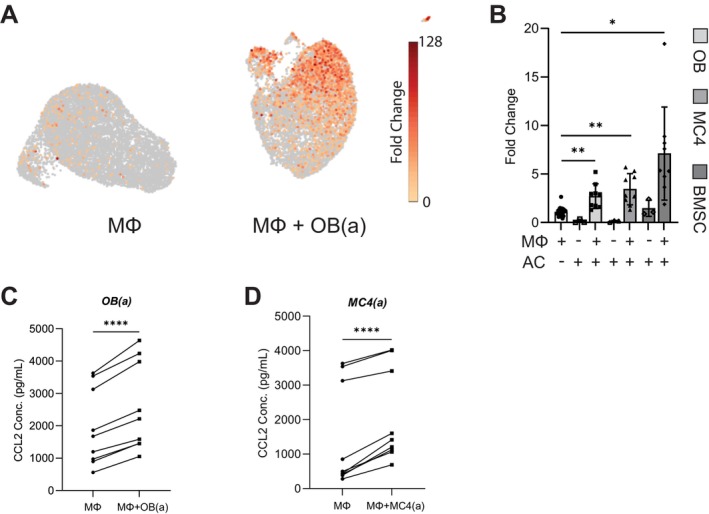
CCL2 expression in efferocytic macrophages. (A) Single‐cell RNA sequencing analysis discriminates efferocytic MΦ (right cluster) from control MΦ (left cluster), with higher *Ccl2* gene expression in efferocytic MΦs. (B) *Ccl2* gene expression via qRT‐PCR in MΦ cocultured for 18 h with apoptotic cells (AC): Primary calvarial osteoblasts (OB), pre‐osteoblasts (MC4), or bone marrow stromal cells (BMSC). (C, D) CCL2 cytokine expression via ELISA in culture media of MΦs co‐cultured with apoptotic (C) osteoblasts (OB(a)) or (D) pre‐osteoblasts (MC4(a)). Data from 3 independent experiments are shown. Statistical significance was evaluated using mixed‐effects analysis, with the Geisser–Greenhouse correction (B) or paired t‐tests (C, D). *p < 0.05, **p < 0.01, ****p < 0.0001.

Macrophages are known to alter their transcriptional activity and secretome following efferocytosis, typically in a manner that supports tissue repair and regeneration. Among the many cytokines expressed by macrophages, changes in *Ccl2* expression are specifically of interest due its known role as a myeloid cell chemoattractant and potential function as a mesenchymal stem/progenitor cell (MSPC) chemoattractant. To examine transcriptional changes as a function of efferocytosis, single‐cell RNA sequencing (scRNA‐seq) was performed using the 10X Genomics platform on naïve macrophages and macrophages engulfing apoptotic osteoblasts (OB(a)). ScRNA‐seq was selected over bulk RNA sequencing for our ongoing work exploring shifts in efferocytic macrophage profiles, including identifying sub‐populations of macrophages unique to efferocytosis. Interestingly, results showed a significant increase (10.6x fold change) in overall *Ccl2* gene expression in efferocytic macrophages compared to naïve macrophages, though only a subset of efferocytic macrophages expressed high levels of *Ccl2* (Figure 1A).

Similar efferocytosis experiments were performed to validate observations from the scRNA‐seq analysis and further examine the dependence of *Ccl2* upregulation on apoptotic cell type. Gene expression determined by qRT‐PCR confirmed that efferocytic macrophages upregulated *Ccl2* expression after engulfing either OB(a) or pre‐osteoblasts (MC4(a)) (Figure 1B). Macrophage engulfment of apoptotic bone marrow stromal cells (BMSC(a)), used as a surrogate for MSPCs, also led to significantly upregulated *Ccl2* expression (Figure 1B). *Ccl2* gene expression was negligible in apoptotic cells (ACs) alone, supporting the finding that efferocytosis regulates macrophage transcriptional activity.

Next, an ELISA was used to quantify CCL2 in media from the aforementioned efferocytosis experiments. In line with gene expression studies, efferocytic macrophages significantly increased CCL2 secretion (Figure 1C,D). Taken together, these results demonstrate that efferocytosis of different types of ACs likely to be present at a bone injury site (osteoblasts, pre‐osteoblasts, MSPCs) consistently leads to the upregulation of CCL2 expression.

### 3.2 CCL2 Acted as a Chemoattractant for MSPCs

**FIGURE 2:**
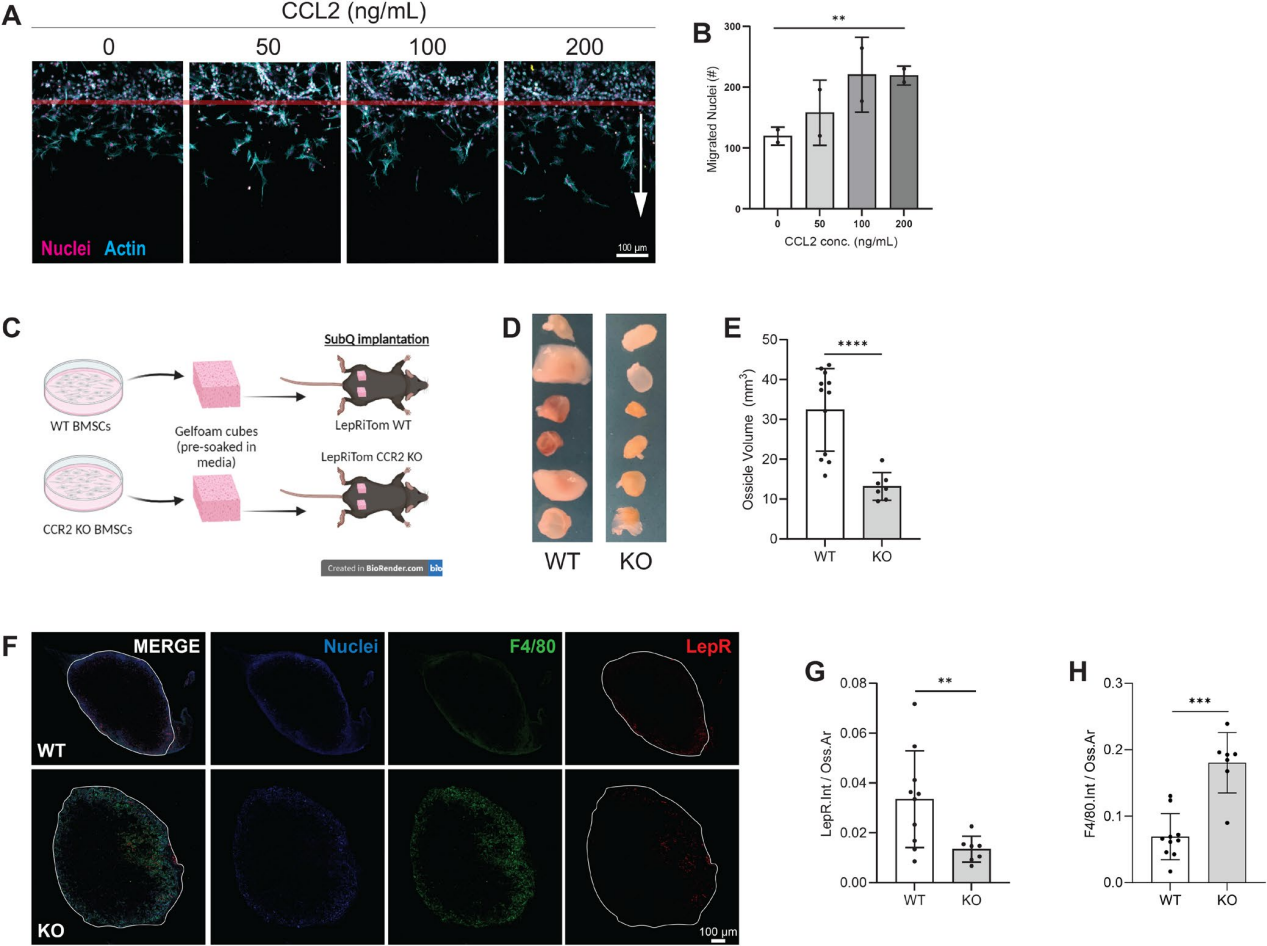
MSPC migration to CCL2 in vitro & in an in vivo ectopic ossicle model (A) 7 day migration of BMSCs to varying doses of CCL2 in a 3 mg/mL collagen type 1 gel. Gels immunostained for Nuclei/Hoechst (pink) and Actin (cyan). Arrow indicates direction of migration. (B) Quantification of migrated cells > 50 μm from channel wall (red line). The representative data set is from one of two independent experiments shown. Statistical significance was evaluated using RM one‐way ANOVA, with the Geisser‐Greehhouse correction and the Dunnett's multiple comparisons test. (C) Representative model of ossicle implantation: WT BMSC‐ or CCR2 KO BMSC‐seeded ossicles were implanted in WT LRT (*n* = 12) and CCR2 KO LRT (*n* = 7) mice, respectively. (D) Representative images of ossicles explanted after 4 weeks and (E) quantification of explanted ossicle volume. (F) Representative images of immunostained ossicles. DAPI (nuclei; blue), F4/80 (green), LepR (red). (G, H) Quantification of (G) F4/80 and (H) LepR intensity normalised to ossicle area. Statistical significance for was evaluated using unpaired t‐tests, with Welch's correction (E, G, H). *p < 0.05, **p < 0.01, ***p < 0.001, ****p < 0.001. LepR = LepR‐Cre^+/−^ Rosa‐tdTomato^+/−^.

To examine the chemotactic influence of CCL2 on non‐myeloid lineage cells, we employed a collagen type 1 hydrogel‐based 3D migration assay. This assay was chosen over more traditional transwell migration assays as migration through a 3D extracellular matrix and adhesion to collagen type 1 more closely mimics the microenvironment encountered by infiltrating MSPCs, namely the collagen‐rich fibrocartilagenous network laid down by infiltrating repair‐mediating cells [38, 39]. A diffusive gradient of CCL2 was imposed on BMSCs (surrogates for MSPCs) and migration through the gel was analysed over 6–7 days. A significantly greater number of BMSCs migrated through the collagen type 1 matrix when exposed to a CCL2 gradient (Figure 2A,B).

Next, we employed a murine ectopic ossicle implantation model to assess CCL2 as an MSPC chemoattract in vivo [32]. In this model, BMSCs were seeded onto gelatin sponges (pre‐soaked in BMSC growth media) and subcutaneously implanted into the backs of mice. These implants develop into bony structures containing cortical and trabecular bone and a bone marrow. This model enables investigation of bone‐cellular interactions with opportunities to assess donor‐derived and host‐derived cell behaviour in response to genetic manipulations. Additionally, this is a relatively rapid in vivo model with multiple possible outcome analyses, including histomorphometry, micro‐CT, and cell tracking via fluorescent reporter proteins [32]. In this study, LepRiTom mice enabling fluorescence‐based lineage tracing of MSPCs were used to elucidate the necessity of CCL2 signalling for endogenous MSPC recruitment. Gelatin sponges seeded with BMSCs from CCR2 KO mice (C5BL/6J background) were implanted into LepRiTom CCR2 KO mice and compared to age‐matched WT/WT controls (i.e., BMSCs from WT mice implanted into LepRiTom WT mice) (Figure 2C).

Ossicles explanted from LepRiTom CCR2 KO mice after 4 weeks were smaller in volume, indicating less cell infiltration in the absence of CCR2 (Figure 2D,E). Furthermore, MSPC infiltration assessed by tdTomato positivity was significantly lower in CCR2 KO ossicles (Figure 2F,G). These results support our earlier in vitro data findings that CCL/R2 signalling acts as a homing signal for MSPCs. Surprisingly, macrophage (F4/80^+^) presence was significantly higher in CCR2 KO ossicles (Figure 2F,H). Further evaluation of WT and CCR2 KO macrophages, via flow cytometry, revealed no significant differences in their efferocytic or phagocytic capability. The percentage of F4/80^+^ macrophages with CFSE^+^ apoptotic pre‐osteoblasts or NileRed^+^ polystyrene microspheres, after 5 h and 25 h of co‐culture, was not significantly different between WT and CCR2 KO macrophages (Figure S1). These data confirm that CCL2 indeed serves as an MSPC chemoattractant (BMSCs in vitro and LepRiTom cells in vivo), while highlighting the complexity of CCL/R2 signalling in regulating macrophage activity and its role beyond serving as a myeloid cell chemoattractant.

### 3.3 *Ccr2* Loss Increases Mineralized Callus During Early Stress Fracture Healing in Male Mice

**FIGURE 3:**
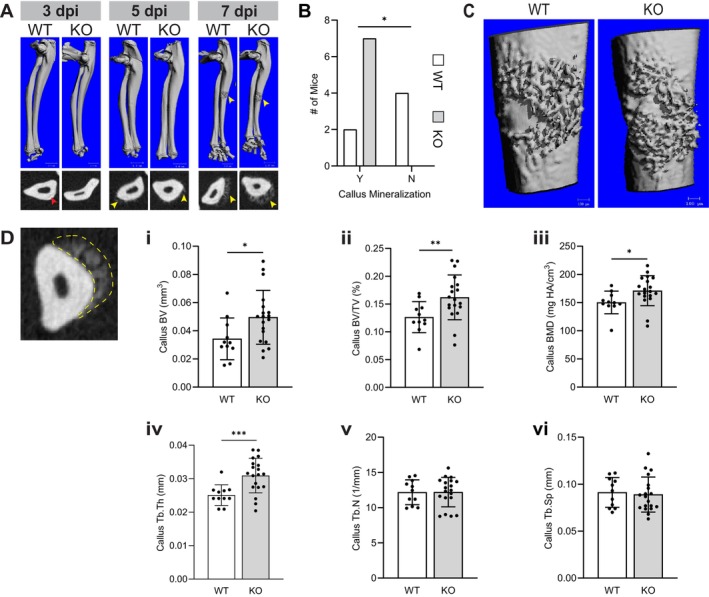
Micro‐CT analysis of mineralized callus in stress fractured (sfx) ulnae. (A) Representative 3D renderings of stress fractured (sfx) ulnae from WT and CCR2 KO mice 3‐, 5‐ and 7‐days post injury (dpi). Below are corresponding x‐rays of the bones at their fracture/callus sites. (B) Incidence of mineralized callus at 5‐dpi. Y is present, N is not present. (C) Representative 3D renderings of 7‐dpi callus on stress fractured ulnae from WT and CCR2 KO mice. (D) Micro‐CT analysis trabecular bone parameters were evaluated for the full length in the callus of WT (*n* = 11) and CCR2 KO (*n* = 19) mice. Callus in x‐ray outlined in yellow. (i) bone volume (BV), (ii) % bone volume (BV/TV), (iii) bone mineral density (BMD), (iv) trabecular number (Tb.N), (v) trabecular thickness (Tb.Th), (vi) trabecular separation (Tb.Sp). Statistical significance was evaluated using Fisher's exact test (B) or unpaired *t*‐tests, with Welch's correction (D).*p < 0.05, **p < 0.01, ***p < 0.001.

An ulnar stress fracture model was selected to investigate early effects in the spontaneous healing response following traumatic bone injury. Cyclical loading of the ulna results in a mid‐diaphyseal stress fracture (sfx) that heals primarily through endochondral ossification. In contrast to ossicle implantation, the sfx occurs in a bone microenvironment comprised of natural macrophage and MSPC niches, enabling study of the endogenous healing response. In these studies, fracture healing was examined at early time points to capture immune and repair cell recruitment. Timing and quality of callus formation were assessed at 3, 5 and 7 days post injury (dpi) in WT and CCR2 KO mice via microCT and IF analysis. Fractured ulnae from both groups lacked calluses at 3‐dpi, but developed mineralized calluses by 7‐dpi (Figure 3A). While many 5‐dpi ulnae had soft calluses, most only displayed faint callus mineralization in x‐rays (Figure 3A). Interestingly, a significantly greater number of CCR2 KO mice had mineralizing calluses at 5‐dpi compared to WT controls (Figure 3B).

Furthermore, micro‐CT analysis of trabecular bone in 7‐dpi calluses revealed CCR2 KO mice had more mineralized calluses compared to WT controls, corresponding to the similar observation seen in 5‐dpi CCR2 KO mice (Figure 3C,D). Calluses in 7‐dpi CCR2 KO mice showed significantly higher total bone volume (BV), bone volume fraction (BV/TV), bone mineral density (BMD), and trabecular thickness (Tb.Th) compared to WT (Figure 3Di–iv). However, there were no significant differences in trabecular number or spacing (Figure 3Dv,vi).

**FIGURE 4:**
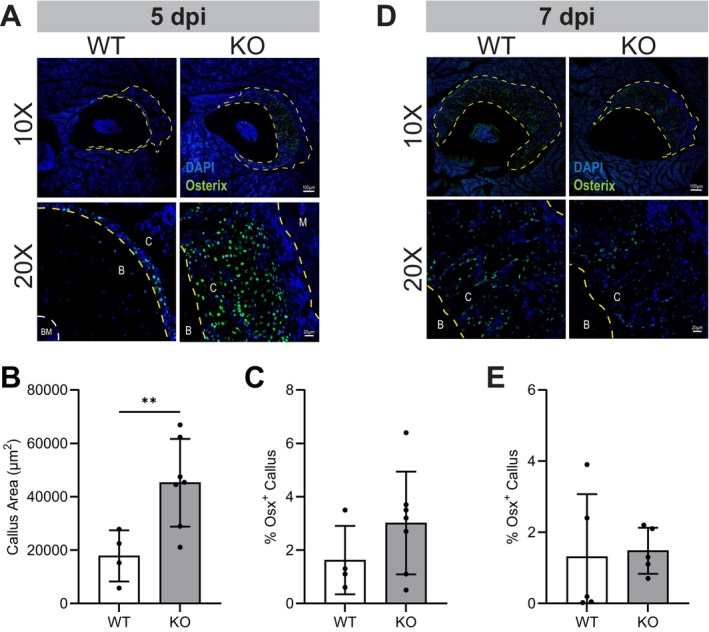
Osteoprogenitor cell infiltration in 5 & 7 dpi stress fractured (sfx) ulnae. Representative confocal images of osterix‐positive (Osx^+^) osteoprogenitor cells in the callus of stress fractured ulnae from WT and CCR2 KO (A) 5‐dpi (*n* = 4 WT, 6 KO) and (D) 7‐dpi (*n* = 5 WT, 5 KO) mice. (B) Quantification of callus area (including soft tissue) at 5‐dpi. (C, E) Quantification of % Osx^+^ cells (fluoresce intensity per callus area) in the callus of (C) 5‐dpi and (E) 7‐dpi mice. Statistical significance was evaluated using unpaired *t*‐tests, with Welch's correction. *p < 0.05, **p < 0.01.

Significant differences in callus mineralization between WT and KO mice prompted further examination of progenitor cell composition of 5‐ and 7‐dpi calluses. To identify osteoprogenitors, cryosections of ulnae containing callus were immunostained for osterix (Osx), a transcription factor expressed in osteogenic progenitor cells. Congruent with the increased presence of mineralized callus seen in micro‐CT x‐rays, total callus area (including soft tissue) within the cryosection was also significantly higher in 5‐dpi CCR2 KO mice compared to WT (Figure 4A,B). The percentage of Osx^+^ cells in the callus or periosteum; however, was not significantly different at 5‐dpi (Figure 4C). There were noticeably fewer Osx^+^ cells at 7‐dpi versus 5‐dpi in calluses, but similar to 5‐dpi, the percentage of Osx^+^ cells was not significantly different between CCR2 KO and WT mice (Figure 4D,E).

## 4 Discussion

Although CCL/R2 signalling has been under investigation for decades, the nuances of this pleiotropic factor's contributions to bone repair are still incompletely understood. This study presents data in support of a role for CCL/R2 signalling in MSPC recruitment and early ulnar stress fracture repair. Upregulation of CCL2 in macrophages efferocytosing bone and bone progenitor cells, a process associated with promoting repair and regeneration, is intriguing given that macrophages engulfing apoptotic neutrophils [18] and apoptotic macrophages (data not shown) did not alter their CCL2 expression. These observations not only indicate that transcriptional regulation in efferocytic macrophages is cargo‐specific, but also further motivates studies of CCL2 given its multiple relevant roles in wound healing including MSPC recruitment.

An in vitro 3D migration assay was used to assess BMSC migration through type I collagen hydrogels in the presence of CCL2 gradients, as progenitor cell recruitment requires migration through the collagen‐rich late‐stage provisional matrix in fracture calluses. BMSC migration in this assay demonstrated a chemotactic response to CCL2, which is supported by other studies investigating MSPC migration outside of the bone microenvironment [24, 40, 41]. For example, Ishikawa et al. reported BMSC migration to CCL2 in a dose dependent manner and inhibition of chemotactic migration by CCR2 blockade [22].

In vivo migration of MSPCs and macrophages was then assessed in a subcutaneous ossicle implantation model, with a primary focus on understanding the impact of CCL/R2 signalling on MSPC migration in vivo, as a follow up to the in vitro MSPC migration study presented earlier. The decrease in LepR^+^ cells within the ossicle supported our in vitro findings that CCL/R2 signalling is involved in MSPC migration. CCL2 is capable of interacting with other receptors such as CCR4, and ACKR1 and ACKR2, although ACKRs do not exhibit chemotactic signalling activity [42]. Similarly, CCR2 has multiple ligands beyond CCL2, including CCL7, CCL8, CCL12, CCL13 and CCL16 [42]. However, CCL2 primarily signals through CCR2 and exhibits significantly higher activity compared to other ligands that also signal through CCR2 [42, 43, 44, 45]. In the current study, CCL/R2 signalling plays a crucial role in MSPC migration, either by directly influencing the ability of MSPCs to home to the ossicle or by modulating other cells that MSPCs interact with for migration cues. The use of a global CCR2 KO model limited our ability to interpret the exact mechanism of reduced MSPC migration as various other cells also respond to CCL/R2 signalling.

Of note, we also found a perplexing increase in F4/80^+^ macrophages within CCR2 KO ossicles. Although CCL2 is a well‐known myeloid cell chemoattractant, CCL/R2 signalling has effects beyond chemotaxis. In fact, CCL/R2 signalling has been associated with promoting myeloid cell survival and proliferation [21]. Reduced or impaired survival and proliferative signalling in myeloid cells may have sustained an apoptotic cell presence in the CCR2 KO ossicles that continually attracted macrophages, which may explain the increased presence of macrophages. Additionally, some studies have shown CCL/R2 signalling is crucial for macrophage endocytosis and degradation of collagen type 1 and fibrin [46, 47]. Thus, ineffective degradation of the gelatin sponges may have also contributed to the prolonged presence of macrophages in CCR2 KO ossicles. Flow cytometry results from efferocytosis of pre‐osteoblasts and phagocytosis of polystyrene beads did not suggest differences in efferocytic or phagocytic ability of WT and CCR2 KO bone marrow derived macrophages. However, other studies found impaired CCL/R2 signalling reduced macrophage phagocytic ability [47, 48]. Cargo‐specificity may explain differences in results as these studies used *E.coli* or dextran to assess phagocytic ability. It is important to note than an increase in macrophage presence does not necessarily equate to increased CCL2 production. Our lab found CCL2 production was specifically elevated in efferocytic macrophages engulfing apoptotic BMSCs (representative of MSPCs) and other bone‐related apoptotic cells (OBs, MC4s), but not apoptotic neutrophils [18] or macrophages (data not shown). Even if macrophage‐associated CCL2 production was elevated in CCR2 KO mice, we would not expect this to aid in recruiting MSPCs to the ossicle as the primary mechanism of CCL2 signalling via CCR2 would be impaired. While alternate mechanisms may explain the unexpected increase in F4/80^+^ macrophages, loss of CCL/R2 signalling negatively affected MSPC infiltration of ossicles as hypothesized.

To determine the impact of CCL/R2 signalling on MSPC recruitment in the context of early bone repair, this study focused on callus formation and early progenitor cell migration in an ulnar stress fracture model. Significantly more CCR2 KO mice had mineralizing calluses by 5‐dpi, and significantly higher BV, BV/TV, BMD, and Tb.Th by 7‐dpi. Wu et al. demonstrated that CCL2 gene and protein expression were significantly upregulated up to 1 day post injury (dpi) in stress fractured rat ulnae compared to unloaded control ulnae, and up to 14‐dpi compared to unloaded contralateral ulnae. Among the factors they investigated (MIP1α, RANTES, CCR1, CCR2, OSM and OSMr), only CCL2 gene expression was significantly upregulated at early time points (4 h, and 1, 4, 7 days) [49]. This suggests that CCL/R2 signalling plays an important role in the early phase of stress fracture repair and remodelling. Although we did not observe any differences in the force or displacement required to induce stress fractures in WT vs. KO ulnae (Figure S2A), other studies have reported stronger tibia in CCR2 KO mice [50, 51]. Mader et al. acknowledged that differences in tibial strength may be in part due to significantly higher body weight of CCR2 KO mice in their study and that differences in CCR2 KO strain development may affect body mass. However, body weight is an unlikely contributor to differences seen here in 7‐dpi ulna stress fracture healing, as the body weight of KO vs. WT mice was significantly lower (25.3 g in KO vs. 27.1 g in WT) in our study (Figure S2B).

Complete fractures typically heal through both endochondral and direct intramembranous ossification, where relative contributions of each process depend on fracture stabilisation and other mechanical forces [52]. Unlike complete fractures, ulnar stress fractures primarily heal via rapid periosteal woven bone formation (intramembranous ossification) around the fracture site [52, 53, 54]. Uthgenannt et al. demonstrated in a rat ulnar stress fracture model partial recovery of ulna strength by 7‐dpi and complete recovery of strength by 14‐dpi, with negligible new bone formation after 7‐dpi. While the woven bone area did not increase, woven bone mineral density increased dramatically (by 80%) between 7‐ and 14‐dpi, in part due to an increase in the mineral: collagen ratio [54]. Others have investigated the effects of impaired CCR2 signalling via genetic knockout or pharmacological inhibition in tibia injury [25, 26, 28, 55], rib fracture [22] and tooth extraction [27] models. Injuries that primarily healed through endochondral ossification reported impaired healing [22, 25, 26] except in elderly mice [55], while those that healed through intramembranous ossification reported no overall impact on bone repair [27, 28]. In contrast, our data shows impaired CCR2 signalling affects bone repair via intramembranous ossification in the ulna stress fracture model. Interestingly, while intramembranous healing in the tibia injury model showed no change in F4/80^+^ cells in CCR2 KO mice 7‐dpi [28], intramembranous healing in the tooth extraction model showed significant decreases in F4/80^+^ cells in alveolar bone at 7‐, 14‐ and 21‐dpi [27]. Moreover, in models which heal via endochondral ossification, studies have reported varied findings. One study observed increased callus volume and new bone formation at 21‐dpi [25], while others reported decreased callus volume 21‐dpi [22], or no difference in callus volume with decreased callus mineralization at 28‐dpi [26]. These discrepancies highlight the variability in outcomes for the same metrics within similar bone repair processes, which may explain why intramembranous healing in ulnar stress fractures differed from that in tibia or alveolar bone injuries. In particular, as ulnar stress fractures appear to dramatically increase callus mineralization density between 7 and 14‐dpi [54], differences in callus bone may be normalised by then. The choice of injury model (location, extent of injury, primary repair mechanism), timing of repair stages, experimental end point, and mouse age at the time of injury (6–14 or 78 weeks vs. 16 weeks in our model) may account for the varied results observed across studies investigating the role of impaired CCL/R2 signalling in fracture healing.

Histological analysis of cryosectioned bone was used to evaluate infiltration of osteogenic cells within the callus. Immunofluorescence analysis of Osx^+^ cells revealed no significant differences in the percentage of osteoprogenitors within the callus at 5‐ or 7‐dpi. In contrast to the 4‐week ossicle implantation model, where LepR^+^ MSPC migration was significantly reduced in KO mice, the ulna stress fracture model showed similar progenitor cell presence at earlier time points (1‐week). Furthermore, the presence of the MSPC and osteoprogenitor niche in the bone marrow, periosteum, and surrounding skeletal muscle in the stress fracture model likely minimised the extent of MSPC migration required to the reach the injury site [52]. These two factors may explain the differing progenitor cell presence observed in CCR2 KO mice between the ossicle and stress fracture models. However, this study was limited by histological processing issues, which prevented the enumeration of other basic multicellular unit (BMU) cells involved in remodelling.

The BMU, a transient unit of cells involved in bone remodelling, consists not only of bone‐forming osteoblasts and bone‐resorbing osteoclasts, but also their progenitor cells, vascular endothelial cells, immune cells (macrophages, neutrophils, T cells) and bone matrix components [56, 57]. Bone remodelling within the BMU is a highly complex process, tightly regulated by local and systemic factors that carefully orchestrate the temporal activation of cells to balance bone anabolism and catabolism. CCL/R2 signalling is likely one of these key regulatory pathways as it influences multiple cell types and processes, including chemotaxis of monocytes/macrophages (OC precursors) [21, 43, 58, 59], MSPCs [23, 24, 60], endothelial cells [58], T cells [21, 58], and neutrophils [26], depending on specific conditions. Interestingly, CCL2 mediated cell migration in a cell‐type dependent manner. For example, monocytic cells relied on Src‐kinases and p38 MAPKs, while endothelial cells depend on Src‐kinases, PI3K, and ERK1/2 but not p38 MAPKs [61]. In the context of bone repair in CCR2 KO models, neutrophils [25, 27] and T cell [27] migration responses were not significantly altered, whereas the migration of F4/80^+^ macrophages [25, 27, 28] varied across studies. Batoon et al. hypothesized that inflammatory macrophages recruited via CCR2 signalling may not be as critical as F4/80^+^ osteal macrophages, which are essential for intramembranous repair [11, 28]. The relative contributions of osteal versus bone marrow‐derived macrophages in bone repair could explain the differences in healing outcomes observed in various CCR2 KO studies [28]. To our knowledge, in vivo MSPC or osteoprogenitor migration in CCR2 KO bone injury models has not been reported by others. In our study, we demonstrated that CCR2 KO does not affect the presence of Osx^+^ osteoprogenitors at 7‐dpi in an ulnar stress fracture model, despite the chemotactic effect of CCL2 observed in vitro.

Osteogenic differentiation of osteoprogenitors (e.g., BMSCs) supplemented with CCL2, as well as osteoblast (OB) markers such as OB surface, mineral apposition rate, and OCN serum levels in uninjured CCR2 KO mice are not significantly different from controls. However, OBs appear to be indirectly affected by CCL/R2 signalling, as observed in mice subjected to alveolar bone injuries supplemented with MCP‐1 [59] or with impaired CCR2 signalling [27]. OBs are considered a major source of CCL2 in bone, particularly in response to inflammatory factors [58]. They also increase CCL2 production via NF‐κB and C/EBP activation when stimulated by PTH [58], and via ERK1/2 activation in response to mechanical loading [60]. In this paper, we propose that efferocytic macrophages represent another significant source of CCL2 production during bone injury.

Osteoclasts (OCs) appear to be most significantly affected by impaired CCL/R2 due to its involvement in all stages of OC development: recruitment of OC precursors (monocyte/macrophage), differentiation/fusion, and resorption capacity [58]. As with other BMU cells, OC presence and activity in CCR2 KO mice vary across bone injury and bone loss models. Some studies reported a decrease [51, 62], while others reported no difference [25] or an increase [27]. Only one study demonstrated decreased OC number, OC surface per bone surface, and size in uninjured CCR2 KO mice [51], while others found no differences in OC numbers [62] or resorption marker levels [50] at baseline. In alveolar bone injury models, OC presence increased in CCR2 KO mice [27, 63]. In MSPC (Prx1^+^)‐ specific CCR2 KO mice, both BMSC‐derived RANKL and OC presence decreased, but only when mice were treated with LPS to induce lung inflammation and subsequent bone loss [62]. This variation suggests the impact of CCL/R2 signalling on OCs is more pronounced during injury, when active bone remodelling is required. CCL/R2 signalling activated NF‐ κB and ERK1/2, but not p38 MAPK or JNK pathways in pre‐osteoclasts, upregulating RANK expression [51], enhancing the effects of RANKL to promote OC fusion [58], and reducing OC resorption capacity [25, 51]. Considering multiple direct and indirect effects of CCL/R2 signalling on OCs, impaired OC recruitment and/or activity may explain the increased callus mineralization observed at 7‐dpi in ulnar stress fractured CCR2 KO mice.

Given the pleiotropic nature of CCL/R2 signalling and its extensive impact on the crosstalk between multiple cells types actively involved in bone remodelling, more comprehensive investigations of cell composition during healing could inform early bone repair interventions. Our study highlights the relevance of CCL/R2 signalling in an apoptotic‐cell rich injury environment, and shows that while MSPCs are recruited via this chemotactic axis, the necessity of this pathway is injury‐model dependent. Future studies into cell infiltration and compensatory mechanisms in early fracture healing could further clarify the differing findings in the ulnar stress fracture model compared to other CCL/R2 impaired intramembranous healing models. Furthermore, using cell‐specific CCR2 KO transgenic mice (e.g., MSPC‐specific LepR^+^) and broader immunofluorescence analyses, particularly in bone injury models associated with common comorbidities such as diabetes and osteoporosis, could provide additional insights into the nuanced roles of CCL/R2 signalling in bone repair.

## Author Contributions

**Rahasudha Kannan:** data curation (lead), formal analysis (lead), funding acquisition (equal), investigation (lead), methodology (equal), project administration (equal), visualization (lead), writing – original draft (lead), writing – review and editing (equal). **Amy J. Koh:** formal analysis (supporting), funding acquisition (supporting), investigation (supporting), methodology (equal), writing – review and editing (equal). **Robert N. Kent III:** formal analysis (supporting), software (supporting). **Kaira Bhutada:** formal analysis (supporting). **Fatima Wasi:** investigation (supporting). **Leon Wagner:** investigation (supporting). **Kenneth Kozloff:** resources (supporting), supervision (supporting), writing – review and editing (supporting). **Brendon M. Baker:** resources (supporting), supervision (supporting), writing – review and editing (supporting). **Hernan Roca:** conceptualization (lead), methodology (supporting), supervision (lead), writing – review and editing (supporting). **Laurie K. McCauley:** conceptualization (lead), funding acquisition (equal), methodology (supporting), project administration (equal), resources (lead), supervision (equal), writing – review and editing (equal).

## Conflicts of Interest

The authors declare no conflicts of interest.

## Supporting information


**Figure S1.** Phagocytic capacity of WT and CCR2 KO macrophages. Flow cytometry analysis of WT or CCR2 KO macrophages (MΦ) cocultured for 5 or 25 h with (A) CFSE‐stained apoptotic pre‐osteoblasts (MC4(a)) fed at a 2:1 MC4(a) to MΦ ratio or (B) fluorescent (Nile Red) 1.7–2.2 μm Spherotech polystyrene microspheres (MS) fed at a 1:1 MS to MΦ ratio. MΦ were stained with F4/80‐APC. Data from 2 independent experiments are shown. Statistical significance was evaluated using mixed‐effects analysis, with the Geisser–Greenhouse correction.

**Figure S2.** CCR2 KO vs. WT stress fractured mice parameters. (A) Total loading force required to fully fracture ulna of WT (*n* = 4) or CCR2 KO (*n* = 5) mice. (B) Body weight (BW) of WT (*n* = 11) or CCR2 KO (*n* = 19) mice used in 7‐dpi stress fracture (sfx). experiments taken BW was measured before inducing sfx. Statistical significance was evaluated using unpaired t‐tests, with Welch’s correction. *p < 0.05.

## Data Availability

The data that support the findings of this study are available from the corresponding author upon reasonable request.
